# Cellular Players in Gastrointestinal Involvement of Systemic Sclerosis: Insights into Pathogenesis

**DOI:** 10.3390/cells14231930

**Published:** 2025-12-04

**Authors:** Silvia Peretti, Francesco Bonomi, Giulia Bandini, Cristiano Barbetta, Michael Hughes, Francesco Del Galdo, Marco Matucci Cerinic, Zsuzsanna H. McMahan, Silvia Bellando Randone

**Affiliations:** 1 Department of Internal Medicine, University Hospital Careggi, 50134 Florence, Italy; silvia.peretti@unifi.it (S.P.); giulia.bandini@unifi.it (G.B.); 2 Scleroderma Unit, Rheumatology Department, Department of Experimental and Clinical Medicine, University of Florence, 50134 Florence, Italy; cristiano.barbetta@unifi.it (C.B.); silvia.bellandorandone@unifi.it (S.B.R.); 3 Centre for Musculoskeletal Research, Division of Musculoskeletal and Dermatological Science, School of Biological Sciences, Faculty of Biological Medicine and Health, The University of Manchester, Manchester Academic Health Science Centre, Manchester M13 9PT, UK; michael.hughes-6@manchester.ac.uk; 4 Department of Rheumatology, Salford Care Organisation, Northern Care Alliance NHS Foundation Trust, Salford M6 8HD, UK; 5 NIHR Manchester Biomedical Research Centre, Manchester University NHS Foundation Trust, Manchester M6 8HD, UK; 6 Leeds Institute of Rheumatic and Musculoskeletal Medicine, University of Leeds, Leeds LS2 9JT, UK; f.delgaldo@leeds.ac.uk; 7 NIHR Biomedical Research Centre, Leeds Teaching Hospitals NHS Trust (LTHT), Leeds LS7 4SA, UK; 8 Unit of Immunology, Rheumatology, Allergy and Rare Diseases (UnIRAR), IRCCS San Raffaele Scientific Institute, 20132 Milan, Italy; matuccicerinic.marco@hsr.it; 9 Vita-Salute San Raffaele University, 20132 Milan, Italy; 10 Inflammation Fibrosis and Ageing Initiative (INFLAGE), Division of Genetics and Cell Biology, IRCCS San Raffaele Scientific Institute, 20132 Milan, Italy; 11 Division of Rheumatology, Department of Internal Medicine, UTHealth Houston, Houston, TX 77030, USA; zsuzsanna.h.mcmahan@uth.tmc.edu

**Keywords:** systemic sclerosis, gastrointestinal involvement, pathogenesis, enteric nervous system, interstitial cells of Cajal, fibroblast activation, epithelial barrier dysfunction, microbiota–immune interactions, endothelial dysfunction, functional autoantibodies

## Abstract

**Background**: Gastrointestinal (GI) involvement is the most frequent visceral complication of systemic sclerosis (SSc), affecting up to 90% of patients, yet it remains poorly understood compared to pulmonary or cutaneous manifestations. The aim of this review is to integrate current knowledge on the cellular mechanisms underlying GI disease in SSc and to identify research priorities. **Methods**: A narrative literature review was conducted through a systematic PubMed search up to September 2025, complemented by manual reference screening. **Results**: Histopathological and functional evidence consistently demonstrates that neuromuscular alterations, including degeneration of enteric neurons, loss of interstitial cells of Cajal, and smooth muscle atrophy, can precede fibrosis, challenging the traditional “fibrosis-first” paradigm. Fibroblast and myofibroblast activation are present in gastric and colonic samples, sustained by profibrotic mediators such as TGF-β, CTGF, and endothelin-1, although the cellular origins of these stromal cells remain uncertain. Additional pathogenic contributions include autonomic dysfunction, barrier dysfunction with dysbiosis, impaired vascular reserve of vessels perfusing the gut, and functional autoantibodies targeting interneural and neuromuscular function and communication. Compared with skin and lung, the GI tract displays less fibrosis and fewer inflammatory infiltrates, but immune-derived mediators and autoantibodies suggest distinct immunopathogenic pathways are activated. **Conclusions**: Collectively, these findings depict GI involvement in SSc as a multi-compartmental process integrating neural, epithelial, endothelial, stromal, and immune alterations. Addressing the lack of validated biomarkers, mechanistic models, and biomarker-stratified trials will be essential to move beyond symptomatic care and toward precision medicine approaches for SSc-related GI disease.

## 1. Introduction

Gastrointestinal (GI) involvement represents the most frequent visceral complication of systemic sclerosis (SSc), affecting up to 90% of patients [1,2,3,4], yet paradoxically remains the least understood at the cellular and mechanistic level. Unlike interstitial lung disease or pulmonary hypertension, where pathogenesis and therapeutic strategies have advanced considerably, the GI tract remains without effective targeted interventions to modify disease progression, despite its profound impact on morbidity, mortality, and quality of life [5]. Clinically, GI involvement in SSc is highly heterogeneous, encompassing gastroesophageal reflux, dysphagia, gastroparesis, small bowel dysmotility, chronic intestinal pseudo-obstruction, diarrhea, constipation, and anorectal dysfunction [2,3]. Despite this broad clinical spectrum, no widely adopted biomarkers currently exist to aid in diagnostic stratification, and evaluation still relies on invasive procedures such as manometry and endoscopy, or on non-invasive but not routinely accessible tests such as scintigraphy [2,3]. Because these assessments are not routinely feasible in all patients, clinical evaluation remains predominantly symptom-driven, often relying on patient-reported outcomes to capture functional burden [2]. Recent large-scale patient surveys have confirmed the disproportionate symptom burden and unmet needs linked to GI disease, emphasizing its central role in health-related quality of life [6]. One major barrier to progress in pathophysiological research has been the pronounced clinical heterogeneity of GI disease. This has now been substantiated by longitudinal evidence, revealing discrete trajectories of GI progression with unique serological and systemic correlates, thereby redefining our view of SSc GI involvement as a spectrum of subsets rather than a uniform complication [7].

Histopathological studies consistently show that neuromuscular alterations, including degeneration of enteric neurons, loss of interstitial cells of Cajal, and smooth muscle atrophy, can precede or occur independently of overt fibrosis, challenging the classical “fibrosis-first” paradigm. This observation, first anticipated by Sjögren’s model in 1994, underscores the potential for an early therapeutic window before structural remodeling develops [8].

Emerging data also highlight the role of functional autoantibodies (e.g., anti-muscarinic M3 receptor and anti-M2/PDC-E2 antibodies), epithelial barrier dysfunction with dysbiosis, and vascular abnormalities such as impaired splanchnic reserve [9,10,11]. These findings point to a complex interplay between neural, epithelial, endothelial, and stromal compartments in driving dysmotility and tissue injury.

In this review, we adopt a cellular lens to examine GI involvement in SSc. By analyzing the contributions of enteric neurons, ICCs, fibroblasts, endothelial cells, epithelial cells, and immune infiltrates, we aim to integrate current knowledge, highlight unresolved questions, and discuss potential cellular pathways that could be explored for therapeutic intervention.

## 2. Materials and Methods

A narrative literature review was conducted through a systematic search of the PubMed database, including all articles published up to 15 September 2025, restricted to those written in the English language. The search strategy broadly combined the terms “systemic sclerosis” AND “gastrointestinal” with keywords relating to specific cellular players of interest (“enteric neurons,” “interstitial cells of Cajal,” “fibroblasts,” “endothelial cells,” “epithelial cells,” “immune cells”). Additional relevant publications were identified through manual screening of reference lists from selected articles and from international consensus documents.

Studies were considered for inclusion based on their relevance to the pathophysiology and clinical management of GI involvement in SSc, and encompassed original research articles, narrative and systematic reviews. Given the narrative design of the review, no formal risk-of-bias assessment or quantitative synthesis was performed.

Given the narrative design of this review, no formal risk-of-bias assessment was performed, as recommended for non-systematic mechanistic reviews. Our aim was to integrate histologic, functional, and translational data relevant to GI involvement in SSc. When discussing cellular mechanisms that are well established in other SSc-affected organs (e.g., skin or lung), we included such evidence only to illustrate biologically plausible pathways, explicitly indicating in the main text when these remain hypothetical or not yet demonstrated in the gastrointestinal tract. This approach allows us to map potential cellular contributors to SSc-related GI disease while maintaining transparency regarding the level of evidence.

## 3. Enteric Nervous System and Interstitial Cells of Cajal

One of the earliest and most disabling features of GI involvement in SSc is dysmotility, which cannot be solely attributed to fibrotic remodeling [12,13,14]. Growing evidence support the pivotal role of autonomic and enteric nervous system (ENS) dysfunction in SSc GI disease [15].

The autonomic regulation of the GI tract relies on a dual-tiered neural control: the extrinsic autonomic nervous system (ANS) and the intrinsic ENS. The ANS exerts top-down modulation from the central nervous system, with the parasympathetic branch (mainly vagus nerve, acetylcholine-mediated) promoting motility, vasodilation and secretion, and the sympathetic branch (norepinephrine-mediated) exerting inhibitory effects on motility and vasoconstriction [16,17,18].

In contrast, the ENS forms an intrinsic, semi-autonomous network embedded within the gut wall, consisting of the myenteric (Auerbach’s) plexus, located between the circular and longitudinal muscle layers, regulating motility and the submucosal (Meissner’s) plexus controlling secretion, absorption, and the microcirculation [19]. The ENS, often referred to as the “second brain” of the GI tract, operates independently of CNS input, containing a full repertoire of neurons, glial cells, neurotransmitters, and pacemaker cells, coordinating complex reflexes such as peristalsis, segmentation, and sphincter relaxation [20]. Key among these intrinsic elements are the interstitial cells of Cajal (ICCs), which integrate electrical and chemical signals within the neuromuscular apparatus.

### 3.1. Interstitial Cells of Cajal: Loss and Smooth Muscle Atrophy

ICCs are c-Kit^+^ (CD117) mesenchymal-derived pacemaker cells that generate and propagate slow-wave activity and mediate neuromuscular transmission within the GI tract [21]. Their survival and function depend critically on stem cell factor (SCF) signaling through the c-Kit receptor. The contribution of ICCs to GI pathology in SSc has been investigated in both the esophagus and the intestine.

Autopsy and histological studies have demonstrated a significant loss of ICCs in SSc. In the esophagus, a case–control autopsy study on 74 scleroderma patients highlighted a ICC reduction, particularly in areas of circular muscle atrophy, suggesting that impaired neuromuscular signaling contributes to myocyte degeneration and motility failure [22]. Similarly, small bowel tissue from SSc patients with severe hypomotility showed disruption of the myenteric ICC network, whereas colonic ICC density appeared relatively preserved [23]. Importantly, in both sites these abnormalities were observed even in the absence of advanced fibrosis, suggesting that ICC and neuronal injury may precede fibrotic remodeling and contribute directly to early dysmotility.

Ultrastructural studies provide a temporal framework for these observations. Deep rectal biopsies from patients with recent-onset SSc revealed axonal degeneration, cytoskeletal abnormalities in unmyelinated fibers, and focal smooth muscle cell (SMC) damage, occurring in the absence of significant fibrosis [24]. In contrast, full-thickness gastric samples obtained from patients undergoing surgery for severe gastroesophageal involvement showed advanced-stage pathology, with marked SMC disarray, ultrastructural damage to enteric nerve fibers, and abundant pericellular collagen and elastin deposition [25,26]. Comparable findings were described in the esophagus, where surgical and autopsy specimens demonstrated extensive fibrotic remodeling closely associated with neuromuscular dysfunction [27]. Together, these data outline a continuum from early autonomic/SMC involvement without fibrosis to late fibrotic remodeling, disrupting ICCs, ENS, and SMCs.

Although ICCs are known to be highly dependent on SCF/c-Kit signaling and susceptible to ischemia or inflammatory injury in other GI disorders [28,29,30], direct evidence of such mechanisms in SSc is currently lacking. Available studies only document ICC loss in areas of smooth muscle atrophy or reduced network density in the small intestine, without demonstrating a causal link to ischemia, oxidative stress, or immune-mediated injury.

### 3.2. Enteric Nervous System: Structural Damage and Autoimmune Targets

In addition to ICC loss and smooth muscle atrophy, alterations of the ENS play a central role in SSc-related GI dysfunction. Histological studies report decreased neuronal density in the myenteric plexus and reduced submucosal nerve fibers in the colon, in some cases correlating with severe dysmotility [23].

Beyond structural changes, several functional autoantibodies targeting enteric neural elements have been identified in SSc (summarized in Table 1) [31,32]. Over two decades ago, a single experimental rat model showed that passive transfer of IgG from an antibody-positive SSc patient induced alterations in intestinal myoelectrical activity, leading to the hypothesis of myenteric neuronal antibodies and supporting a potential, though unconfirmed, functional role of these antibodies in GI dysmotility [33]. With time, more specific antigenic targets within the myenteric plexus have been characterized; among these, anti-muscarinic M3 receptor (M3R) antibodies are the most extensively studied [34]. M3R are predominantly expressed on GI smooth muscle cells but are also present on enteric neurons. Functional assays have shown that sera from SSc patients containing anti-M3R antibodies preferentially bind neuronal elements and inhibit smooth muscle contractility, indicating that impaired neuronal signaling contributes to motility failure [35]. A recent large cohort study confirmed that approximately 40% of SSc patients harbor anti-M3R antibodies, with high-titre positivity associated with diffuse cutaneous disease, clinically severe GI involvement and anti-RNPC3 (U11/U12) and/or U1RNP positivity [13,36]. Anti-RNPC3 antibodies have likewise been associated with moderate-to-severe GI disease (Medsger GI ≥ 2: 36% vs. 15%, *p* < 0.01), more frequent esophageal dysmotility (93% vs. 62%, *p* < 0.01), and increased odds of significant GI involvement (Medsger score ≥ 2; OR 3.8, 95% CI 1.0–14.3) [13,36]. Their co-occurrence with anti-M3R suggests the possibility of a shared pathogenic pathway: muscarinic signaling can modulate ribonucleoproteins involved in RNA processing, linking altered cholinergic tone with RNA dysregulation and the emergence of specific autoantibody profiles [37]. Notably, anti-RNPC3 and anti-U1RNP positivity have also been associated with more significant pulmonary fibrosis, indicating a more fibrotic systemic phenotype [13]. Another autoantibody associated with more severe GI disease in SSc is anti-gephyrin, detected in approximately 9% of SSc patients [38]. Gephyrin is a scaffolding protein necessary for the clustering of GABA-A and glycine receptors at inhibitory synapses; its targeting may explain symptoms such as bloating and constipation, likely through impaired inhibitory ENS signaling.

Less common but mechanistically intriguing are antimitochondrial M2 antibodies (AMA-M2), directed against the E2 subunit of the pyruvate dehydrogenase complex (PDC-E2) located on the inner mitochondrial membrane and are classically associated with primary biliary cholangitis. In SSc, these were recently shown to correlate with delayed esophageal transit and gastric emptying [39], and higher anti-M2 antibody titers correlated with worse GI motility. Furthermore, in vitro studies demonstrated their ability to penetrate enteric neurons, localize to mitochondria, and impair mitochondrial respiration [39].

Furthermore, anti-vinculin antibodies have been observed at higher levels in SSc compared with healthy controls and correlated with greater GI symptom burden (GI-VAS, *p* < 0.0001) [40,41,42]. Vinculin is a cytoskeletal protein linking actin to the cell membrane, essential for cell adhesion and GI motility [43,44]. Recent scintigraphy-based data further support this association, showing that anti-vinculin antibody positivity—detected in approximately 23% of SSc patients—is significantly associated with slower gastric transit, with higher antibody titers inversely correlating with percent gastric emptying (β = −3.6, *p* = 0.04) [42].

Finally, a recent study on 130 SSc patients identified the presence of antibodies against the cytolethal distending toxin (anti-CDT), a bacterial byproduct produced by *Campylobacter jejuni* and other Gram-negative bacteria, in 18% of cases. Anti-CDT positivity was strongly associated with GI symptoms, particularly fecal soilage, as well as with spliceosome-targeting autoantibodies (RNPC3 and U1RNP). The overlap between anti-CDT and these RNA-processing autoantibodies may support a model of infection-triggered autoimmunity, in which molecular mimicry between microbial and self-antigens contributes to enteric neural injury and SSc-related GI disease [45].

**Table 1:** Autoantibodies associated with GI involvement in SSc. GI, gastrointestinal; SSc, systemic sclerosis; anti-M3R, anti-muscarinic acetylcholine receptor type 3 antibody; anti-RNPC3, anti-RNA-binding region-containing protein 3 antibody; anti-U1RNP, anti-U1 small nuclear ribonucleoprotein antibody; anti-gephyrin, anti-gephyrin antibody; AMA-M2, anti-mitochondrial antibody type M2; anti-CDT, anti-cytolethal distending toxin antibody; PDC-E2, pyruvate dehydrogenase complex E2 subunit; PBC, primary biliary cholangitis.

Autoantibody	Target Antigen/Localization	Associated GI Manifestations	Associated Clinical and Immunologic Features	Proposed Pathogenic Mechanism	Key References
**Anti-M3R**	Muscarinic M3 receptor (smooth muscle & enteric neurons)	Severe GI involvement; dysmotility	Diffuse cutaneous SSc, anti-RNPC3 and/or U1RNP positivity	Blockade of cholinergic signaling → impaired smooth muscle contraction and neuronal transmission	[13,33,34,35]
**Anti-RNPC3 (U11/U12)**	Ribonucleoprotein complex involved in RNA splicing	Moderate-to-severe GI disease (Medsger GI ≥ 2), esophageal dysmotility	Diffuse SSc; pulmonary fibrosis; anti-M3R and anti-U1RNP co-positivity	RNA-processing dysfunction; possible cross-talk with cholinergic pathway	[13,35,36]
**Anti-U1RNP**	Small nuclear ribonucleoprotein	Severe GI and pulmonary fibrosis when co-positive with anti-RNPC3	Overlap or mixed connective tissue disease features	Dysregulation of RNA metabolism and fibrosis pathways	[13,36]
**Anti-gephyrin**	Cytoskeletal scaffolding protein at inhibitory synapses (GABA-A, glycine receptors)	Bloating, severe constipation	No specific associated extra-intestinal features	Impaired inhibitory ENS signaling → dysmotility	[37]
**Antimitochondrial M2 (AMA-M2)**	PDC-E2 (pyruvate dehydrogenase complex, inner mitochondrial membrane)	Delayed esophageal transit, gastric emptying	Sometimes overlap with PBC	Antibody internalization → mitochondrial dysfunction, impaired mitochondrial respiration	[38]
**Anti-vinculin**	Cytoskeletal protein linking actin to membrane (cell adhesion, motility)	Slower gastric emptying; high GI-VAS	n/a	Impaired cytoskeletal dynamics and neuromuscular transmission	[40,41,42,43]
**Anti-CDT**	Cytolethal distending toxin (Campylobacter jejuni byproduct)	Fecal soilage, GI symptoms	Co-occurrence with anti-RNPC3, anti-U1RNP	Infection-triggered molecular mimicry leading to enteric neural autoimmunity	[45]

## 4. Fibroblasts and Myofibroblasts: Core Effectors of Fibrosis

Fibroblasts and myofibroblasts are well recognized as central mediators of fibrosis in skin and lung in SSc, yet their role in the GI tract is less clearly defined.

**Figure 1:**
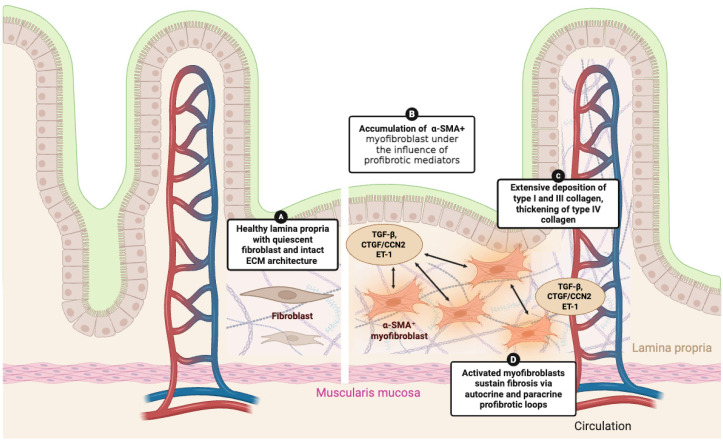
**Fibroblast activation and extracellular matrix remodeling in the gastrointestinal tract of systemic sclerosis.** Illustration of fibroblast-to-myofibroblast transition and extracellular matrix changes in SSc. In healthy tissue (**A**), quiescent fibroblasts maintain an organized ECM architecture. Under the influence of profibrotic mediators such as TGF-β, CTGF/CCN2, and ET-1 (**B**), fibroblasts differentiate into α-SMA^+^ myofibroblasts, leading to excessive deposition of type I and III collagen and thickening of type IV collagen (**C**). Activated myofibroblasts perpetuate fibrosis through autocrine and paracrine profibrotic loops (arrows, (**D**)), contributing to irreversible stromal remodeling in the GI tract.

Histological analyses from full-thickness and endoscopic gastric samples from SSc patients have revealed extensive deposition of type I and III collagen within the lamina propria, thickening of type IV collagen around glands and small vessels, and accumulation of α-SMA^+^ myofibroblasts [46]. These structural changes are paralleled by increased expression of profibrotic mediators, including Transforming Growth Factor-β (TGF), Connective Tissue Growth Factor/Cellular Communication Network Factor 2 (CTGF/CCN2), and endothelin-1 (ET-1), suggesting the presence of autocrine and paracrine circuits that sustain fibroblast activation [46] (Figure 1).

Functional studies provide complementary insights. Colonic myofibroblasts isolated from SSc patients display a constitutively activated phenotype, with elevated α-SMA, COL1A1, and CTGF expression, enhanced migratory capacity, and resistance to apoptosis [47]. Their fibrogenic activity is amplified by thrombin/PAR-1 and ET-1/ETA signaling but can be significantly reduced in vitro through pharmacologic inhibition of these pathways [47]. Together, these findings support the concept of a persistent profibrotic program driving irreversible remodeling in the SSc gut [46,47,48].

The origins of GI myofibroblasts remain uncertain. Mechanisms such as endothelial-to-mesenchymal transition (EndoMT), epithelial-to-mesenchymal transition (EMT), pericyte activation, and recruitment of bone marrow-derived fibrocytes have been well described in other SSc-affected organs (e.g., lung) and are scientifically plausible in the gut, particularly under chronic TGF-β stimulation and ischemia [49]. However, these processes have not yet been directly demonstrated in GI tissue and currently remain speculative.

Overall, fibroblasts represent a central but incompletely characterized effector population in SSc GI involvement. Current data are limited to gastric and colonic samples, with little information on other segments of the GI tract. Defining the cellular origins, molecular pathways, and therapeutic vulnerabilities of these stromal cells remains a major research priority.

## 5. Intestinal Epithelial Cells: Barrier Dysfunction and Microbiota Interactions

Histopathological and functional studies indicate that epithelial alterations are a key feature of GI involvement in SSc. In the stomach, biopsies reveal profound epithelial changes with increased apoptosis, reduced proliferation, and glandular atrophy, which closely correlate with disease severity [50]. These structural abnormalities are paralleled by expansion of myofibroblast populations and collagen deposition, ultimately resulting in loss of mucosal function [50].

In the small intestine, earlier studies based on jejunal biopsies reported only minimal abnormalities [51], however more recent work has revealed that epithelial injury is in fact common [52]. Duodenal biopsies show partial villous atrophy, crypt hyperplasia, increased intraepithelial lymphocytes, and dense mononuclear infiltrates in the lamina propria, pointing to an active epithelial–immune crosstalk [52].

**Figure 2:**
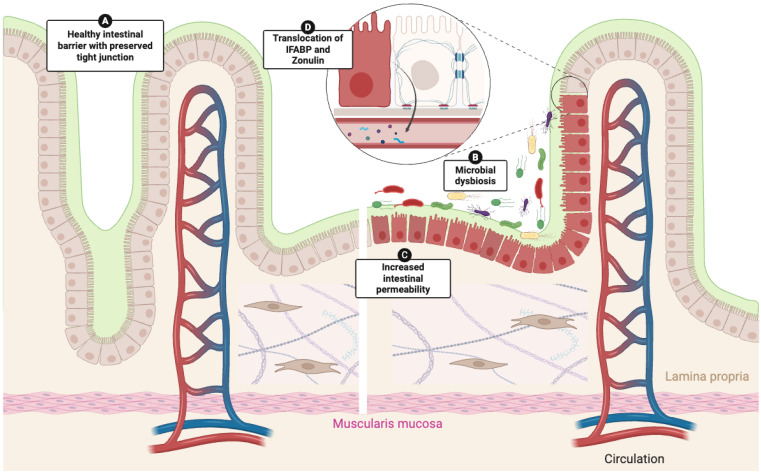
**Intestinal epithelial barrier dysfunction and microbial dysbiosis in systemic sclerosis.** Schematic representation of the intestinal barrier in health (**A**) and systemic sclerosis (**B**–**D**). In SSc, microbial dysbiosis contributes to increased intestinal permeability (**C**), resulting in translocation of epithelial injury markers such as intestinal fatty acid-binding protein (I-FABP) and zonulin (arrow, (**D**)). These alterations promote barrier disruption, mucosal immune activation, and sustained epithelial–microbiota crosstalk.

Biomarker studies further support the presence of functional epithelial damage (Figure 2). Elevated circulating intestinal fatty acid binding protein (I-FABP), a marker of enterocyte injury, together with increased zonulin, which regulates tight junction disassembly, provide indirect evidence of altered epithelial turnover and junctional instability [53]. In parallel, increased lipopolysaccharide-binding protein (LBP) and reduced endotoxin core antibodies (EndoCab) IgM levels suggest microbial translocation [53]. Importantly, these alterations correlate with the severity of GI symptoms, reinforcing the link between epithelial barrier dysfunction, immune activation, and clinical burden [53].

Barrier impairment is further amplified by microbial dysbiosis (Figure 2). Several cross-sectional studies have shown reproducible alterations in the gut microbiota of patients with SSc, particularly in those with overt GI symptoms. These include depletion of commensals with anti-inflammatory properties such as *Faecalibacterium*, *Clostridium*, and *Roseburia*, alongside the expansion of pathobionts including *Fusobacterium*, *Prevotella*, *Streptococcus*, and *Lactobacillus* [54,55,56]. Functional implications of microbiota–epithelial interactions are suggested by a randomized placebo-controlled pilot trial of fecal microbiota transplantation (FMT), in which patients treated with anaerobically cultivated human intestinal microbiota (ACHIM) experienced reductions in bloating, diarrhea, and fecal incontinence, accompanied by shifts in IgA-coated bacterial taxa [57]. Collectively, these findings suggest that dysbiosis not only accompanies but may exacerbate epithelial barrier disruption and sustain mucosal inflammation.

Overall, the epithelial phenotype in SSc appears to be dominated by barrier dysfunction and immune–microbiota crosstalk. Although EMT is a recognized profibrotic mechanism in SSc skin and lung [58], no direct evidence supports its occurrence in the GI tract. This suggests that epithelial cells contribute to GI involvement primarily through barrier failure and mucosal immune interactions rather than through transdifferentiation into myofibroblasts.

## 6. Endothelial Cells and Pericytes: Vascular Pathology

Microvascular dysfunction is a central feature of SSc, and although direct mechanistic data from the GI tract remain limited, accumulating evidence indicates that it also contributes significantly to GI involvement.

**Figure 3:**
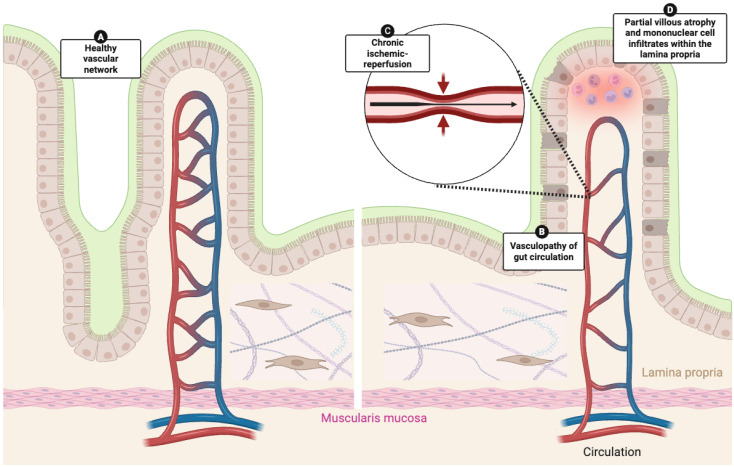
**Vascular dysfunction and ischemia–reperfusion injury in the gastrointestinal tract of systemic sclerosis.** Illustration showing progression from a normal vascular network (**A**) to vasculopathy of the gut circulation (**B**), characterized by chronic ischemia–reperfusion injury (arrows, (**C**)) leading to partial villous atrophy, mononuclear cell infiltrates, and mucosal inflammation within the lamina propria (**D**). These features reflect diffuse microangiopathy extending from systemic to gastrointestinal tissues.

Direct evidence from the GI tract comes from hemodynamic and histopathological studies. Doppler ultrasound has demonstrated a reduced fasting mesenteric blood flow in SSc patients compared with healthy controls, indicating impaired splanchnic vascular reserve and early endothelial dysfunction of the gut circulation [59,60]. Complementary observations from gastric and duodenal biopsies reveal partial villous atrophy, mononuclear cell infiltrates within the lamina propria, and mucosal inflammatory changes consistent with chronic ischemia–reperfusion injury (Figure 3) [52,61,62].

Histopathological analyses of gastric tissue have also demonstrated capillary dilatation, endothelial swelling and detachment, subendothelial collagen deposition, and narrowing of vascular lumina, findings consistent with chronic ischemic injury and local microangiopathy [26]. Endothelial activation with upregulation of adhesion molecules such as VCAM-1, ICAM-1, and E-selectin, accompanied by perivascular CD4^+^ lymphocytic infiltration, further supports a sustained endothelial–immune interaction within the gastric wall [27]. Ultrastructural studies have described endothelial loss, pericyte rarefaction, and disorganization of the capillary network in the muscular and submucosal layers, pointing to diffuse microvascular remodeling [25].

Clinically, SSc-related vascular alterations are frequently associated with GI vascular lesions such as gastric antral vascular ectasia (GAVE), telangiectasias, and intestinal angiodysplasias [63,64]. A videocapsule study on 50 patients demonstrated that these lesions correlate with extra-digestive vasculopathy including digital ulcers and severe nailfold capillaroscopy abnormalities, supporting the concept of a diffuse microangiopathy affecting both skin and GI mucosa. However, a direct causal relationship has not been demonstrated, and mechanistic studies are warranted to clarify the underling pathophysiology [65].

Mechanistic insights into endothelial pathology are largely derived from studies in skin, lung, and systemic vessels, rather than directly from the GI tract. Endothelial cells in SSc are prone to apoptosis, in part mediated by anti-endothelial cell antibodies, and exhibit dysregulated production of vasoactive mediators such as nitric oxide (NO), prostacyclin, and endothelin-1 (ET-1) [64,66]. The latter is of particular interest because it promotes vasoconstriction while also directly stimulating fibroblast and smooth muscle activation, thereby linking vascular and fibrotic pathology. Another process of potential relevance is EndoMT, whereby endothelial cells lose vascular markers and acquire mesenchymal traits, possibly contributing to the pool of fibroblast-like cells. However, as previously said, while EndoMT has been documented in dermal and pulmonary tissues, its occurrence in the GI tract remains speculative [64,66].

In summary, studies in SSc patients provide evidence of GI endothelial dysfunction, including reduced splanchnic vascular flow and the presence of vascular lesions such as GAVE, telangiectasias and angiodysplasias, which are thought to represent morphological consequences of underlying systemic microangiopathy. While endothelial apoptosis, vasoactive imbalance, and EndoMT have been more deeply documented in other organs, their contribution to GI pathology remains to be demonstrated.

## 7. Immune Cells: Inflammation and Immune–Fibrotic Crosstalk

Compared with skin and lung, evidence of immune involvement in the GI tract of SSc is currently relatively limited [67,68,69]. Histopathological studies of esophageal, gastric, small bowel and colon typically show predominant neuromuscular alterations and, in some cases, fibrotic remodeling rather than dense inflammatory infiltrates [22,23,46].

Nevertheless, selected immune-related changes have been documented at the tissue level. In gastric biopsies, immunohistochemistry revealed markedly reduced adiponectin expression compared with controls [70]. Adiponectin is an adipocyte-derived cytokine (adipokine) with potent anti-inflammatory, anti-fibrotic, and vasculoprotective functions. It regulates macrophage polarization, inhibits TGF-β-driven fibroblast activation, and promotes endothelial homeostasis. Reduced circulating and tissue adiponectin levels have been observed in SSc and linked to vascular dysfunction and skin fibrosis, suggesting a systemic metabolic–immune imbalance. In the gastric wall, the loss of adiponectin expression may thus reflect local amplification of these systemic profibrotic processes. Adiponectin-positive areas were enriched in CD4^+^ T cells, whereas adiponectin-negative regions showed a higher prevalence of CD68^+^ macrophages, indicating a shift from a regulatory to a macrophage-dominated pro-fibrotic microenvironment. Such local deficiency of adiponectin may favor persistent macrophage activation, extracellular matrix deposition, and tissue fibrosis, thereby connecting metabolic dysregulation with immune-mediated remodeling in the GI tract [70].

Beyond these local findings, systemic biomarkers further implicate immune pathways in GI pathology. Serum levels of IL-40, a newly described B cell-associated cytokine, was found to be elevated in SSc patients compared with controls, with levels correlating significantly with GI symptoms assessed by the UCLA GIT 2.0 instrument [71]. In vitro, IL-40 stimulates peripheral blood mononuclear cells from SSc patients to release IL-6, MCP-1, and IL-10, indicating that it could amplify immune activation relevant to GI dysfunction [71].

Taken together, although the GI tract in SSc does not usually harbor dense immune infiltrates, emerging evidence suggests that immune-derived mediators, ranging from adipokines to B cell-related cytokines, may contribute to epithelial and stromal alterations. This pattern suggests a distinct tissue-specific pathogenic trajectory, where immune–fibrotic interactions in the GI tract differ from those observed in skin or lung.

## 8. Expert Perspective: Gaps and Future Directions

**Figure 4:**
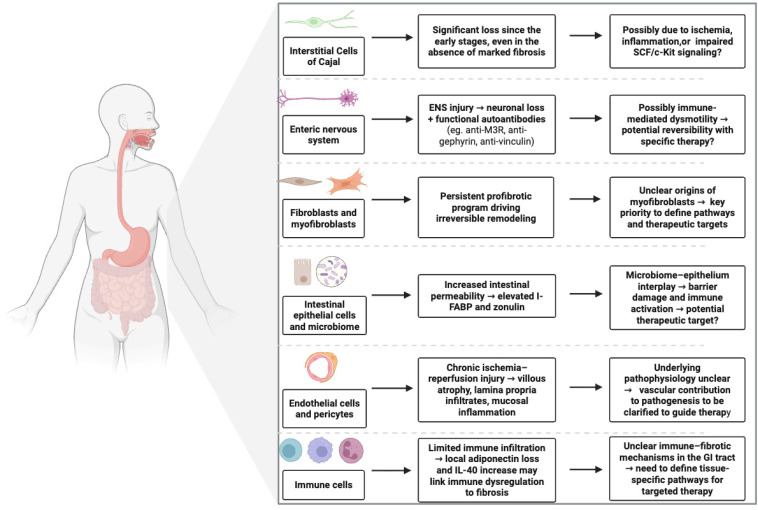
**Cellular players in gastrointestinal involvement of systemic sclerosis.** Overview of the major cellular components implicated in SSc-related GI disease. Interstitial cells of Cajal, enteric neurons, fibroblasts, epithelial cells, endothelial cells, pericytes, and immune cells contribute through distinct yet interconnected mechanisms including neuronal loss, profibrotic activation, epithelial barrier damage, vascular remodeling, and immune dysregulation. Persistent crosstalk among these compartments sustains tissue remodeling and defines potential therapeutic targets.

Despite the high prevalence and clinical burden of GI involvement in SSc, major knowledge gaps in underlying pathobiology persist. An overview of the main proposed pathogenic mechanisms is provided in Figure 4. The absence of reliable animal models hampers mechanistic studies, particularly those addressing neuroimmune dysregulation and epithelial–stromal–fibrotic interactions. Validated biomarkers reflecting disease activity are still lacking. Candidate autoantibodies, including anti-M3R, anti-fibrillarin, anti-RNPC3, anti-M2, anti-gephyrin, anti-CDT, and anti-vinculin, may aid in risk stratification. However, several remain exploratory and require further validation, particularly in their potential to reflect disease activity. Similarly, the cellular origin of GI myofibroblasts is undefined: fibroblast activation, epithelial- and endothelial-to-mesenchymal transition, and fibrocyte recruitment are all plausible but unproven within the GI tract. The contribution of the epithelium and microbiota also remains poorly characterized, with preliminary evidence suggesting barrier dysfunction and altered neuroimmune crosstalk as dominant features. Finally, there is a striking lack of therapeutic trials specifically targeting GI disease, and current ACR/EULAR 2024 recommendations do not support immunosuppression due to insufficient mechanistic and biomarker data [72]. The absence of validated biomarkers and understanding of GI disease mechanisms limits our ability to define disease activity, optimal timing of immunosuppression, and treatment discontinuation strategies [73].

Beyond these gaps lies a broader challenge: understanding why SSc affects the GI tract in such a heterogeneous manner. Distinct functional patterns suggest that immune responses selectively target specific cellular lineages, contributing to variable clinical phenotypes. Emerging evidence points to lineage-specific vulnerabilities, for instance, mitochondrial-rich mesodermally derived enteric neurons targeted by anti-M2/PDC-E2 antibodies, which may drive GI dysfunction and link visceral and systemic manifestations. We propose that differential targeting of GI cell subsets, including ICC, enteric neurons, epithelial cells, and stromal populations, underlies the clinical diversity of SSc. ICC dysfunction may disrupt pacemaker activity and motility, whereas neuronal or stromal injury may manifest as pseudo-obstruction, dysphagia, or vascular dysregulation. Importantly, these cell types or their analogues exist across organs, providing scientific rationale and implying that SSc pathogenesis may stem from shared lineage-specific susceptibilities throughout the body.

The next frontier in understanding SSc-related GI disease will require the integration of three complementary dimensions: (1) mapping the specificity and timing of autoimmune and neuroimmune responses that drive early neuromuscular injury, (2) defining cell-type-specific vulnerabilities within enteric neurons, ICCs, epithelial, endothelial, and stromal populations, and (3) elucidating how microbial and metabolic environmental factors modulate these cellular interactions and disease progression. This framework offers a path toward defining biologically meaningful disease subsets, identifying predictive biomarkers, and developing targeted therapies. By elucidating the mechanisms that drive selective cell injury and systemic propagation, we can move toward interventions that not only halt disease progression but also restore function and improve outcomes in patients with SSc.

Several factors should be taken into account when interpreting this review. As a narrative review, it did not include a formal risk-of-bias assessment, and the selection of studies was designed to integrate key histologic, functional, and mechanistic evidence rather than to provide a comprehensive systematic synthesis. Furthermore, the available literature on GI involvement in SSc remains limited and uneven across different segments of the GI tract, and our synthesis necessarily reflects the scarcity of direct experimental and histopathological data. This knowledge gap represents one of the major unmet needs in the field and may also imply that pathogenic mechanisms vary across distinct GI segments, an aspect that current evidence is insufficient to resolve. The lack of robust GI-specific studies underscores the need for dedicated mechanistic and biomarker-driven research. Finally, in areas where direct GI data were sparse, we incorporated mechanistic insights such as EndoMT, EMT, or fibrocyte recruitment derived from other SSc-affected organs; these pathways were included only when biologically plausible and are explicitly marked in the text as hypothetical in the absence of GI-specific confirmation.

## 9. Conclusions

GI involvement in SSc represents a complex, multi-compartmental disease process that extends far beyond fibrosis. Histologic and functional studies consistently reveal early neuromuscular alterations, including smooth muscle atrophy, loss of ICC, and ENS injury that may precede overt fibrotic remodeling. Stromal activation and fibroblast-driven architectural changes are evident, yet the cellular origins and molecular circuits remain incompletely defined. At the epithelial interface, apoptosis, barrier dysfunction, and microbial dysbiosis link luminal factors to mucosal and stromal responses, while vascular contributions manifest through impaired splanchnic perfusion and ischemic complications. Although immune infiltrates are often sparse, functional autoantibodies and immune-derived mediators add further layers of dysregulation.

These findings underscore that SSc-GI disease should not be considered as a secondary fibrotic complication but a manifestation of coordinated dysfunction across neural, epithelial, stromal, vascular, and immune compartments. Importantly, many of these cell types have analogs in other organ systems, suggesting that GI pathology may reflect broader systemic mechanisms. A deeper understanding of cell-type-specific vulnerabilities and their molecular underpinnings is urgently needed to identify tractable therapeutic targets, develop functional biomarkers, and enable precision, organ-specific interventions.

## Data Availability

No new data were created or analyzed in this study. Data sharing is not applicable to this article.
